# Characteristics and risk factors for inconsistency between the risk of exacerbations and the severity of airflow limitation in COPD based on GOLD 2017: A retrospective, cross-sectional study

**DOI:** 10.1371/journal.pone.0193880

**Published:** 2018-03-12

**Authors:** Wei-Chang Huang, Ming-Feng Wu, Hui-Chen Chen, Jeng-Yuan Hsu, on behalf of The Taiwan Obstructive Lung Disease Consortium

**Affiliations:** 1 Department of Internal Medicine, Division of Chest Medicine, Taichung Veterans General Hospital, Taichung, Taiwan; 2 Department of Medical Technology, Jen-Teh Junior College of Medicine, Nursing and Management, Miaoli, Taiwan; 3 Department of Life Sciences, National Chung Hsing University, Taichung, Taiwan; 4 Department of Medical Laboratory Science and Biotechnology, Central Taiwan University of Science and Technology, Taichung, Taiwan; 5 Department of Medical Research, Division of Clinical Research, Taichung Veterans General Hospital, Taichung, Taiwan; 6 School of Medicine, China Medical University, Taichung, Taiwan; 7 Department of Physical Therapy, Chung-Shan Medical University, Taichung, Taiwan; National and Kapodistrian University of Athens, GREECE

## Abstract

**Background and objectives:**

The clinical implications of the discordance between the risk of exacerbations and the level of airflow limitation in patients with chronic obstructive pulmonary disease (COPD) are still unknown. This study aimed to clarify the clinical significance of such discordance in the management of COPD by exploring its characteristics and risk factors.

**Methods:**

In this retrospective, cross-sectional study, participating physicians completed a detailed patient record form for each participating outpatient with COPD. The data, collected by the Taiwan Obstructive Lung Disease consortium, were managed and analyzed.

**Results:**

Of the enrolled participants, 316 (41.7%) had an inconsistency between the risk of exacerbations and the severity of airflow limitation. Univariate analysis showed that more severe airflow limitation (p = 0.000), higher COPD assessment test (CAT) scores (p = 0.003) and modified Medical Research Council (mMRC) scales (p = 0.008), and the presence of at least one (p = 0.000) or two (p = 0.003) co-morbidities were significantly associated with such inconsistency. More severe airflow limitation (Global Initiative for Chronic Obstructive Lung Disease (GOLD) 3 and 4 classification; odds ratio (OR) = 27.09, p = 0.000 and OR = 25.15, p = 0.000, respectively) and the presence of at least one co-morbidity (OR = 2.01, p = 0.001) were still associated with the inconsistency in multivariate logistic regression analysis. Furthermore, the presence of wheezing (OR = 3.90, p = 0.000) and at least two co-morbidities (OR = 5.43, p = 0.005) were independent risk factors for an inconsistency of a high risk of exacerbations / GOLD 1 or 2 and the CAT score≧10 (OR = 1.58, p = 0.007), mMRC scale 2–4 (OR = 1.53, p = 0.017), and the presence of at least one co-morbidity (OR = 2.55, p = 0.000) for an inconsistency of a low risk of exacerbations / GOLD 3 or 4.

**Conclusions:**

The patients with COPD and an inconsistency between the risk of exacerbations and level of airflow limitation had unique clinical characteristics and risk factors for this inconsistency. Management of these patients should include more detailed evaluations.

## Introduction

As described previously [1], the complexities of chronic obstructive pulmonary disease (COPD) mean that comprehensive assessments are required for its management. The Global Initiative for Chronic Obstructive Lung Disease (GOLD) committee provided a two-dimensional assessment tool that takes into account both exacerbation risk and symptom assessment to allow for the appropriate treatment of COPD in 2014 [2]. In this tool, the risk of exacerbations of COPD is determined by both a history of exacerbations in the previous one year and forced expiratory volume in one second (FEV1) % predicted.

Emerging evidence indicates that the FEV1 by itself is a poor predictor of exacerbations and mortality in patients with COPD [3,4]. Therefore, the GOLD committee defined the future risk of exacerbations solely on the history of exacerbations in the previous one year in 2017 [5]. This change in definition of the risk of exacerbations in COPD from GOLD 2014 to GOLD 2017 has resulted in inconsistencies between the risk of exacerbations and severity of airflow limitation, in that the patients with COPD with a low risk of exacerbations may have severe to very severe airflow limitation (FEV1 < 50%), whereas those with a high risk of exacerbations may have mild to moderate airflow limitation (FEV1 ≥ 50%). However, the clinical implications of the discordance between the risk of exacerbations and level of airflow limitation are unknown.

We hypothesized that these inconsistencies may have a significant clinical impact. Therefore, the aim of this study was to clarify the clinical significance of such inconsistencies in the management of COPD by exploring their characteristics and risk factors.

## Materials and methods

### Study design, setting and population

The design, setting and population of this study had been reported in detail elsewhere [1]. Briefly, this large-scale, cross-sectional, multi-center, observational, retrospective study invited patients with COPD diagnosed according to GOLD 2011 recommendations and fulfilling the inclusion and exclusion criteria to participate in at the outpatient services of 12 teaching hospitals throughout Taiwan between November 2012 and August 2013 [6]. For the study purpose, patients were further excluded if they did not have a detailed history of exacerbations in the previous one year (including prior hospitalizations) to classify the risk of exacerbations, or spirometry results to define the level of airflow limitation based on GOLD 2017 [5]. This study was approved by the individual Institutional Review Boards and Ethics Committees of Chiayi Chang Gung Memorial Hospital, Cheng-Hsin General Hospital, Far Eastern Memorial Hospital, Mackay Memorial Hospital, National Taiwan University Hospital, Taipei Tzu Chi Hospital, Linkou Chang Gung Memorial Hospital, China Medical University Hospital, Taichung Veterans General Hospital, Chia-Yi Christian Hospital, Kaohsiung Chang Gung Memorial Hospital, and E-DA Hospital (approval number: CE13164), and written informed consent was provided by each participant.

### Data collection

The detailed methods of data collection are available elsewhere [1]. Summarily, participating physicians recorded baseline characteristics, COPD-related clinical data, comorbidities of interest, and maintenance pharmacological treatments defined as that continuously prescribed in the previous 3 months before enrollment for each participant from medical records at a single study visit.

### Exacerbation risk

As described in detail previously [1], an exacerbation was defined as a worsening of symptoms that required antibiotics or systemic steroids, emergency room visits or hospitalizations. Based on GOLD 2017 [5], a history of ≥ 2 exacerbations within one year and/or a history of at least one hospitalization due to exacerbation in the preceding year were used to define a high risk of exacerbations rather than the GOLD spirometric classification with GOLD 3 and GOLD 4. The other patients were defined as having a low risk of exacerbations.

### COPD patient group

According to GOLD 2017 [5], the participants were classified into four groups (A, B, C or D) according to their COPD symptoms as determined by the COPD assessment test (CAT) or the modified Medical Research Council dyspnea scale (mMRC) and the risk of exacerbations as defined above. If there was a discrepancy between the symptom assessment tools, the tool with the highest risk was used.

### Consistency between the risk of exacerbations and level of airflow limitation

The participants were categorized into two study groups (consistency or inconsistency) according to the risk of exacerbations and GOLD classification of airflow limitation severity based on GOLD 2017 [5]. The patients with a low risk of exacerbations and GOLD 1 or 2, and those with a high risk of exacerbations and GOLD 3 or 4 were classified into the consistency group. The other patients were classified into the inconsistency group.

### Statistical analysis

All data were expressed as mean and standard deviation for continuous variables or number (percentage) for categorical variables. Comparisons were conducted using the independent *t*-test for continuous variables and chi-square test for categorical variables. A logistic regression model was used to analyze potential factors associated with inconsistencies between the risk of exacerbations and severity of airflow limitation if significant in univariate analysis. Statistical significance was set at *p*<0.05. Statistical analysis was performed using SPSS version 18.0 (SPSS Inc., Chicago, IL, USA).

## Results

**Fig 1:**
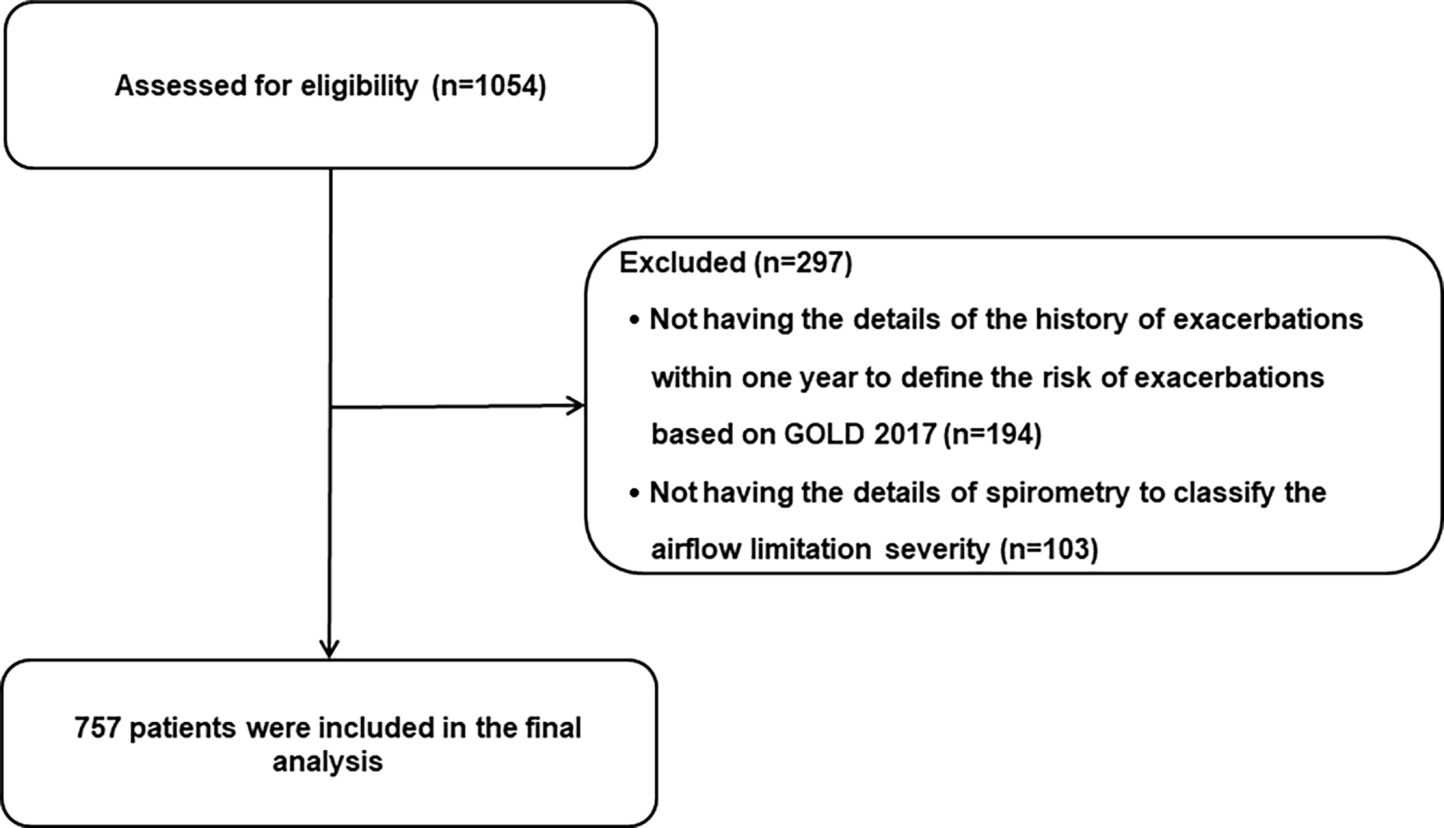
Patient enrollment flow chart. Abbreviations: GOLD, the Global initiative for Chronic Obstructive Lung Disease.

Fig 1 shows the patient enrollment flow chart. This observational study included a total of 757 subjects who fulfilled the inclusion and exclusion criteria.

Table 1 shows the baseline information of the enrolled participants. Of all participants (n = 757), 176 (23.2%), 435 (57.5%), 24 (3.2%) and 122 (16.1%) subjects were classified into groups A, B, C, and D, respectively, based on GOLD 2017. Furthermore, 728 (96.2%) were male and 235 (31.0%) had a positive bronchodilator test (BT). Of the enrolled participants, 316 (41.7%) had an inconsistency between the risk of exacerbations and the severity of airflow limitation, including 54 (30.7%), 204 (46.9%), 13 (54.2%), and 45 (36.9%) in groups A, B, C, and D, respectively.

**Table 1:** Baseline characteristics of the enrolled patients.

	A (n = 176)	B (n = 435)	C (n = 24)	D (n = 122)	Total (n = 757)
**Age (years)**	70.3±9.4	72.7±9.5	69.8±8.5	73.5±8.9	72.2±9.4
<60	19 (10.8%)	42 (9.7%)	3 (12.5%)	3 (2.5%)	67 (8.9%)
60–69	64 (36.4%)	110 (25.3%)	7 (29.2%)	35 (28.7%)	216 (28.5%)
70–79	66 (37.5%)	159 (36.6%)	11 (45.8%)	52 (42.6%)	288 (38.0%)
≧80	27 (15.3%)	124(28.5%)	3(12.5%)	32 (26.2%)	186 (24.6%)
**Male gender**	168 (95.5%)	422 (97.0%)	24 (100%)	114 (93.4%)	728 (96.2%)
**Smoking**					
Never	11 (6.3%)	36 (8.3%)	1 (4.2%)	12 (9.8%)	60 (7.9%)
Ex-smoker	99 (56.3%)	253 (58.2%)	14 (58.3%)	77 (63.1%)	443 (58.5%)
Current smoker	66 (37.5%)	146 (33.6%)	9 (37.5%)	33 (27.0%)	254 (33.6%)
**BMI**	23.6±3.3	23.4±3.9	22.9±3.6	22.2±3.6	23.2±3.7
**Presence of wheezing**	53 (30.1%)	183 (42.1%)	14 (58.3%)	89 (73.0%)	339 (44.8%)
**Spirometry (Post-bronchodilator test)**					
FEV1/ FVC (%)	57.6±9.0	55.0±9.5	54.9±9.6	50.7±10.0	54.9±9.7
FEV1 (L)	1.5±0.5	1.3±0.5	1.3±0.4	1.0±0.4	1.3±0.5
FVC (L)	2.6±0.8	2.3±0.7	2.3±0.6	2.0±0.6	2.3±0.7
FEV1% predicted	61.9±22.2	54.6±21.9	56.6±19.7	46.8±16.9	55.1±21.6
**GOLD spirometric classification**					
I	40 (22.7%)	51 (11.7%)	4 (16.7%)	6 (4.9%)	101 (13.3%)
II	82 (46.6%)	180 (41.4%)	9 (37.5%)	39 (32.0%)	310 (41.0%)
III	42 (23.9%)	160 (36.8%)	9 (37.5%)	59 (48.4%)	270 (35.7%)
IV	12 (6.8%)	44 (10.1%)	2 (8.3%)	18 (14.8%)	76 (10%)
**Positive bronchodilator test**^**§**^	56 (31.8%)	141 (32.4%)	7 (29.2%)	31 (25.4%)	235 (31.0%)
**CAT scores**	5.2±2.4	11.8±7.1	5.3±2.7	14.9±7.9	10.6±7.2
≧10	0 (0.0%)	263 (60.5%)	0 (0.0%)	94 (77.0%)	357 (47.2%)
**mMRC**	0.8±0.4	2.2±0.7	0.9±0.3	2.5±0.9	1.9±0.9
2–4	0 (0.0%)	370 (85.1%)	0 (0.0%)	107(87.7%)	477 (63.0%)
**Number of exacerbations in the previous year**	0.2±0.4	0.2±0.4	1.9±1.0	2.4±1.5	0.6±1.1
0–1	176 (100%)	435 (100%)	9 (37.5%)	34 (27.9%)	654 (86.4%)
≧2	0 (0.0%)	0 (0.0%)	15 (62.5%)	88 (72.1%)	103 (13.6%)
**Severe exacerbations**	0 (0.0%)	0 (0.0%)	15 (62.5%)	82 (67.2%)	97 (12.8%)
**Inconsistency between the risk of exacerbations and level of airflow limitation**	54 (30.7%)	204 (46.9%)	13 (54.2%)	45 (36.9%)	316 (41.7%)
**Inhaled pharmacological therapy**					
None	18 (10.2%)	39 (9.0%)	1 (4.2%)	10 (8.2%)	68 (9.0%)
LAMA alone	42 (23.9%)	129 (29.7%)	9 (37.5%)	21 (17.2%)	201 (26.6%)
LABA alone	12 (6.8%)	17 (3.9%)	1 (4.2%)	4 (3.3%)	34 (4.5%)
LABA + LAMA	13 (7.4%)	27 (6.2%)	0 (0.0%)	9 (7.4%)	49 (6.5%)
LAMA + ICS	1 (0.6%)	13 (3.0%)	0 (0.0%)	2 (1.6%)	16 (2.1%)
ICS/LABA	43 (24.4%)	97 (22.3%)	2 (8.3%)	38 (31.1%)	180 (23.8%)
ICS/LABA + LAMA	47 (26.7%)	113 (26.0%)	11 (45.8%)	38 (31.1%)	209 (27.6%)
**Methylxanthines**	137(77.8%)	317 (72.9%)	15 (62.5%)	94 (77.0%)	563 (74.4%)
**Co-morbidities**					
Cardiovascular Disease^#^	55 (31.3%)	101 (23.2%)	7 (29.2%)	28 (23.0%)	191 (25.2%)
Chronic lung disease^※^	23 (13.1%)	31 (7.1%)	3 (12.5%)	8 (6.6%)	65 (8.6%)
Lung cancer	3 (1.7%)	6 (1.4%)	0 (0.0%)	5 (4.1%)	14 (1.8%)

Abbreviations: BMI, body mass index; FEV1, forced expiratory volume in one second; FVC, forced vital capacity; GOLD, the Global initiative for Chronic Obstructive Lung Disease; CAT, COPD assessment test; mMRC, modified Medical Research Council dyspnea scale; LAMA, long-acting muscarinic antagonist; LABA, long-acting β2-agonist; ICS, inhaled corticosteroid.

^§^Positive bronchodilator test was defined as FEV 1 or FVC improvement from pre-dose value by ≥ 12% and ≥ 200 mL.

^#^Cardiovascular disease included ischemic heart disease, heart failure, atrial fibrillation and hypertension.

^※^Chronic lung disease included previous pulmonary tuberculosis, bronchiectasis and pneumoconiosis.

We evaluated the characteristics and independent risk factors associated with the inconsistency between the risk of exacerbations and severity of airflow limitation using univariate and multivariate logistic regression analyses. Table 2 shows that a more severe airflow limitation, higher CAT and mMRC scale scores, and the presence of at least one or two co-morbidities were associated with an inconsistency between the risk of exacerbations and the severity of airflow limitation. Further, the presence of wheezing and at least two co-morbidities were associated with an inconsistency of a high risk of exacerbations / GOLD 1 or 2 while the higher CAT and mMRC scale scores and the presence of at least one or two co-morbidities were associated with an inconsistency of a low risk of exacerbations / GOLD 3 or 4.

**Table 2:** Univariate analysis of the demographic characteristics and clinical data of the enrolled patients.

	Consistency (n = 441)	Inconsistency					
		A high risk of exacerbations / GOLD 1 or 2 (n = 58)	p-value	A low risk of exacerbations / GOLD 3 or 4 (n = 258)	p-value	Total (n = 316)	p-value
**Age (years)**^**†**^	72.4±9.4	75.1±8.9	0.051	71.1±9.4	0.080	71.9±9.4	0.417
<60	38 (8.6%)	1 (1.7%)		28 (10.9%)		29 (9.2%)	
60–69	123 (27.9%)	14 (24.1%)		79 (30.6%)		93 (29.4%)	
70–79	165 (37.4%)	25 (43.1%)		98 (38.0%)		123 (38.9%)	
≧80	115 (26.1%)	18 (31.0%)		53 (20.5%)		71 (22.5%)	
**Male Gender**^**‡**^	422 (95.7%)	306 (96.8%)	0.732	251 (97.3%)	0.385	306 (96.8%)	0.538
**Smoking**^**‡**^			0.180		0.429		0.205
Never	32 (7.3%)	6 (10.3%)		22 (8.5%)		28 (8.9%)	
Ex- smoker	250 (56.7%)	38 (65.5%)		155 (60.1%)		193 (61.1%)	
Current smoker	159 (36.1%)	14 (24.1%)		81 (31.4%)		95 (30.1%)	
**BMI**^**†**^	23.5±3.7	23.0±3.4	0.383	22.9±3.8	0.075	22.9±3.8	0.063
**Presence of wheezing**^**‡**^	185 (42.0%)	42 (72.4%)	0.000*	112 (43.4%)	0.766	154 (48.7%)	0.076
**GOLD spirometric classification**^**‡**^							
			0.001*		0.000*		0.000*
I	91 (20.6%)	10 (17.2%)		0 (0.0%)		10 (3.2%)	
II	262 (59.4%)	48 (82.8%)		0 (0.0%)		48 (15.2%)	
III	68 (15.4%)	0 (0.0%)		202 (78.3%)		202 (63.9%)	
IV	20 (4.5%)	0 (0.0%)		56 (21.7%)		56 (17.7%)	
**Positive bronchodilator test**^**‡**^^**§**^	132 (29.9%)	15 (25.9%)	0.627	88 (34.1%)	0.288	103 (32.6%)	0.483
**CAT scores≧10**^**‡**^	187 (42.4%)	32 (55.2%)	0.089	138 (53.5%)	0.006*	170 (53.8%)	0.003*
**mMRC 2–4**^**‡**^	260 (59.0%)	37 (63.8%)	0.573	180 (69.8%)	0.006*	217 (68.7%)	0.008*
**Number of exacerbations in the previous year**^**‡**^			0.000*		0.000*		0.452
0–1	377 (85.5%)	19 (32.8%)		258 (100.0%)		277 (87.7%)	
≧2	64 (14.5%)	39 (67.2%)		0 (0.0%)		39 (12.3%)	
**Severe exacerbations**^**‡**^	62 (14.1%)	35 (60.3%)	0.000*	0 (0.0%)	0.000*	35 (11.1%)	0.271
**Inhaled pharmacological therapy**^**‡**^			0.561		0.204		0.233
None	49 (11.1%)	6 (10.3%)		13 (5.0%)		19 (6.0%)	
LAMA alone	115 (26.1%)	14 (24.1%)		72 (27.9%)		86 (27.2%)	
LABA alone	20 (4.5%)	1 (1.7%)		13 (5.0%)		14 (4.4%)	
LABA + LAMA	32 (7.3%)	2 (3.4%)		15 (5.8%)		17 (5.4%)	
LAMA + ICS	10 (2.3%)	0 (0.0%)		6 (2.3%)		6 (1.9%)	
ICS/LABA	101 (22.9%)	16 (27.6%)		63 (24.4%)		79 (25.0%)	
ICS/LABA + LAMA	114 (25.9%)	19 (32.8%)		76 (29.5%)		95 (30.1%)	
**Methylxanthines**^**‡**^	325 (73.7%)	48 (82.8%)	0.183	190 (73.6%)	1.000	238 (75.3%)	0.675
**Co-morbidities**^**‡**^							
≧1	104 (23.6%)	21 (36.2%)	0.054	113 (43.8%)	0.000*	134 (42.4%)	0.000*
≧2	10 (2.3%)	(8.8%)	0.020*	17 (6.6%)	0.008*	22 (7.0%)	0.003*

*p<0.05 as compared to the consistency group

Abbreviations: see Table 1

^†^independent t test

^‡^chi-square test

^§^see Table 1

Table 3 shows the results of the multivariate logistic regression analysis incorporating all significant factors in the univariate analysis in Table 2. More severe airflow limitation (GOLD 3 and 4 classification) and the presence of at least one co-morbidity were still associated with an inconsistency between the risk of exacerbations and the severity of airflow limitation. Furthermore, the presence of wheezing and at least two co-morbidities was the independent risk factor for an inconsistency of a high risk of exacerbations / GOLD 1 or 2; the CAT score≧10, mMRC scale 2–4, and the presence of at least one co-morbidity for an inconsistency of a low risk of exacerbations / GOLD 3 or 4.

**Table 3:** Logistic regression analysis of significant factors in univariate analysis for all patients.

Independent risk factor for inconsistency	Odds ratio (95% CI)	p value
**A high risk of exacerbations / GOLD 1 or 2 and a low risk of exacerbations / GOLD 3 or 4**		
**GOLD spirometric classification:**		
II vs. I	1.71 (0.82, 3.55)	0.153
III vs. I	27.09 (13.09, 56.07)	0.000*
IV vs. I	25.15 (10.72, 59.02)	0.000*
**CAT scores:** ≧10 vs. <10	1.05 (0.64, 1.47)	0.899
**mMRC:** 2–4 vs. 0–1	0.97 (0.64, 1.47)	0.897
**Co-morbidities**		
≧1 vs. 0	2.01 (1.32, 3.05)	0.001*
≧2 vs. 0	1.99 (0.73, 5.41)	0.177
**A high risk of exacerbations / GOLD 1 or 2**		
**Wheezing:** presence vs. absence	3.90 (2.10, 7.25)	0.000*
**Co-morbidities:** ≧2 vs. 0	5.43 (1.67, 17.69)	0.005*
**A low risk of exacerbations / GOLD 3 or 4**		
**CAT scores:** ≧10 vs. <10	1.58 (1.13, 2.22)	0.007*
**mMRC:** 2–4 vs. 0–1	1.53 (1.08, 2.18)	0.017*
**Co-morbidities**		
≧1 vs. 0	2.55 (1.79, 3.63)	0.000*
≧2 vs. 0	1.92 (0.83, 4.49)	0.130

*p<0.05

Abbreviations: CI, confidence interval; also see Table 1.

## Discussion

### Main findings

This study demonstrated that a significant proportion of the patients with COPD overall and in each GOLD group had an inconsistency between the risk of exacerbations and the severity of airflow limitation. In addition, such inconsistency was associated with more severe airflow limitation, higher CAT and mMRC scale scores, and more comorbidities. Furthermore, more severe airflow limitation (GOLD 3 and 4 classification) and the presence of at least one co-morbidity were independent risk factors for an inconsistency between the risk of exacerbations and the severity of airflow limitation; the presence of wheezing and at least two co-morbidities for an inconsistency of a high risk of exacerbations / GOLD 1 or 2; the CAT sccore≧10, mMRC scale 2–4, and the presence of at least one co-morbidity for an inconsistency of a low risk of exacerbations / GOLD 3 or 4.

### Interpretation of the findings in relation to previously published work

This is the first study to characterize patients with COPD and an inconsistency between the risk of exacerbations and severity of airflow limitation. The results showed that, compared to those with consistency between the risk of exacerbations and severity of airflow limitation, the patients with an inconsistency had more severe airflow limitation, higher CAT and mMRC scale scores, and more comorbidities. Thus, an inconsistency between the risk of exacerbations and severity of airflow limitation has a predictable clinical behavior and may be proposed as a significant clinical phenotype of COPD characterized by more respiratory symptoms, worse lung function and health status, and more comorbidities, especially when other complex disease parameters such as the airway inflammation status reflected by sputum and blood eosinophil counts, airway microbiology, and radiologic characterization are not taken into account, making it easier to use in clinical practice.

Although worsening lung function has been associated with an increased frequency of exacerbations and hospitalizations [7], we found that more severe airflow limitation (GOLD 3 and 4 classification) was an independent predictor of the inconsistency between the risk of exacerbations and severity of airflow limitation in COPD. In other words, patients with COPD and worse lung function probably have a low rate of exacerbations, which is consistent with the results reported in previous studies indicating that the occurrence of exacerbations varies widely, and that FEV1 by itself is not sufficient to predict exacerbations in COPD [3,4,8,9].

COPD is a heterogeneous respiratory disease, and comorbidities can occur to the same extent irrespective of GOLD spirometric grading [10]. These comorbidities have been associated with a higher risk of hospitalization and mortality [11], and they need to be treated. Thus, comorbidities should be identified and managed for each patient with COPD [5], especially for those with an inconsistency between the risk of exacerbations and severity of airflow limitation, for which at least one comorbidity was an independent risk factor in the current study.

A comprehensive assessment of symptoms is recommended for all patients with COPD [5]. We found that, the presence of wheezing was a predictor for patients with COPD and an inconsistency of a high risk of exacerbations / GOLD 1 or 2. Meanwhile, the CAT score≧10 and mMRC scale 2–4 were independent risk factors for patients with COPD and an inconsistency of a low risk of exacerbations / GOLD 3 or 4. This indicates that there are different symptom assessments predictive of these two inconsistencies. Thus, different symptom assessment and therapeutic strategies may be required for these two study subgroups.

Similar with one previous study [12], we found a significant proportion of patients with COPD had an airway reversibility after excluding subjects with a history of asthma. Although the evidence shows that patients with COPD with bronchodilator responsiveness have a 17±4 ml per year greater rate of decline in FEV1 compared to those with a negative BT [13], a body of evidences, along with our findings, indicate a positive BT cannot predict an inconsistency between the risk of exacerbations and severity of airflow limitation, a discordance in COPD group assignment classified based on the CAT score and mMRC scale, the response to long-acting bronchodilator treatment or disease progression [1,14–16]. Thus, for the management of COPD, the clinical implications of airway reversibility require to be studied further.

### Implications for future research, policy and practice

Previous studies have focused on patients with a marked discordance between the severity of airflow limitation and symptoms assessed by CAT or mMRC and suggested that more detailed evaluations should be carried out to identify other factors responsible for such discordances [5]. However, little is known about the clinical implications of inconsistencies between the risk of exacerbations and severity of airflow limitation in patients with COPD. In this study, we found that such inconsistencies may represent a unique clinical phenotype of COPD. Future studies are needed to validate the effects of these inconsistencies on the management and outcomes of patients with COPD. We also found that the comorbidities and symptoms should be recognized and addressed rigorously and comprehensively in patients with COPD, especially in those with an inconsistency between the risk of exacerbations and severity of airflow limitation, as they may require different therapeutic strategies in addition to the current GOLD recommendations.

### Strengths and limitations of this study

As mentioned in detail previously [1], the strengths of this study include that it was performed by qualified pulmonologists actively involved in COPD management throughout Taiwan. In addition, in order to comply with the GOLD 2011 strategy [6], the number of exacerbations was recorded in the preceding one year. We also performed spirometry based on the American Thoracic Society Statement at all of the study institutes [17]. More importantly, an acute worsening of respiratory symptoms without any treatment or treated with short-acting bronchodilators only was not recorded as an exacerbation, making overestimation of risk of exacerbations and inconsistency between the risk of exacerbations and severity of airflow limitation less possible. We believe that our study design was rigorous and could therefore compensate for the limitations of the study described elsewhere [1]. These include that rather than recording all COPD-related co-morbidities, we only recorded the co-morbidities of interest including cardiovascular diseases, chronic lung diseases and lung cancer. Thus, the association between COPD-related co-morbidities and the studied inconsistency in the present study could not be evaluated comprehensively. In addition, the participants were not sampled randomly, and the patients with COPD with a worse health status and respiratory capacity (e.g. CAT score ≥ 30 and mMRC scale score = 4) were less willing to participate in the study which may have led to underestimations of the effects of overall CAT and mMRC scale scores on the studied inconsistencies. Moreover, with regards to differences in assessments of the risk of exacerbations according to the history of exacerbations between GOLD 2011 and GOLD 2017 [5,6], the former defined a high risk when the patients had ≥ 2 exacerbations within one year regardless of a history of hospitalizations due to exacerbations, and the latter considered patients with ≥ 2 exacerbations or a history of hospitalizations in the preceding year to indicate a high risk of exacerbations. As mentioned above, the Taiwan Obstructive Lung Disease study was initially implemented in compliance with GOLD 2011, therefore, some of the participants who had only one exacerbation in the previous one year before enrollment were excluded from this study due to a lack of information as to whether or not this exacerbation led to a hospitalization. Finally, the participants of the present study were composed of only 29 (3.8%) female subjects. Chronic diseases have a variable impact on men and women due to the biologic, physiologic, and sociologic differences. For this reason, our findings may not be applicable to female patients with COPD.

## Conclusions

The patients with COPD and an inconsistency between the risk of exacerbations and level of airflow limitation had unique clinical characteristics and risk factors for such inconsistencies. In addition to the patients with a discordance between the severity of airflow limitation and perceived symptoms, patients with COPD and inconsistencies between the risk of exacerbations and level of airflow limitation should undergo more detailed evaluations.
